# A comparison of diagnostic tests for lactose malabsorption - which one is the best?

**DOI:** 10.1186/1471-230X-9-82

**Published:** 2009-10-31

**Authors:** Øistein Hovde, Per G Farup

**Affiliations:** 1 Department of Medicine, Innlandet Hospital Trust, Gjøvik, Norway; 2 Unit for Applied Clinical Research, Norwegian University of Science and Technology, Trondheim, Norway

## Abstract

**Background:**

Perceived milk intolerance is a common complaint, and tests for lactose malabsorption (LM) are unreliable. This study assesses the agreement between diagnostic tests for LM and describes the diagnostic properties of the tests.

**Methods:**

Patients above 18 years of age with suspected LM were included. After oral intake of 25 g lactose, a combined test with measurement of serum glucose (s-glucose) and hydrogen (H2) and methane (CH4) in expired air was performed and symptoms were recorded. In patients with discrepancies between the results, the combined test was repeated and a gene test for lactose non-persistence was added. The diagnosis of LM was based on an evaluation of all tests. The following tests were compared: Increase in H2, CH4, H2+CH4 and H2+CH4x2 in expired air, increase in s-glucose, and symptoms. The agreement was calculated and the diagnostic properties described.

**Results:**

Sixty patients were included, seven (12%) had LM. The agreement (kappa-values) between the methods varied from 0.25 to 0.91. The best test was the lactose breath test with measurement of the increase in H2 + CH4x2 in expired air. With a cut-off level < 18 ppm, the area under the ROC-curve was 0.967 and sensitivity was 100%. This shows that measurement of CH4 in addition to H2 improves the diagnostic properties of the breath test.

**Conclusion:**

The agreement between commonly used methods for the diagnosis of LM was unsatisfactory. A lactose breath test with measurement of H2 + CH4x2 in expired air had the best diagnostic properties.

## Background

The population based prevalence of lactose malabsorption (LM) in Scandinavia is 2-8% [1-3]. The prevalence of LM is in the same order in subjects with functional gastrointestinal disorders (FGID) as in the general population, whereas perceived milk and lactose intolerance is reported by 30-67% of subjects with FGID [2,4-7]. Dietary advice to subjects with FGID and to those with intolerance to milk and lactose relies on a valid and reliable diagnostic test for LM. Such a test is not available.

Lactose is an unabsorbable disaccharide hydrolysed by lactase (lactase-phlorizin hydrolase) in the intestinal brush border into galactose and glucose that are absorbed. LM is a failure to hydrolyse lactose. In adults the most common cause is a genetic defect with lack of intestinal lactase. The unabsorbed lactose is metabolised by colonic bacteria to produce gas (hydrogen (H2) and methane (CH4)) and short chain fatty acids. Clinical manifestations of LM are abdominal pain/discomfort, borborygmi, bloating, flatulence and diarrhoea [2,8]. In subjects with LM, symptoms depend on the amount of lactose. Most people with LM can, due to colonic adaptation to regular lactose ingestion, ingest up to 6-12 g lactose (120 - 240 mL milk) without developing symptoms [9-11].

Commonly used tests for the diagnosis of LM are based on an exaggerated increase in H2 and/or CH4 in the expired air after intake of lactose, or an inappropriate increase in serum glucose (s-glucose). Other tests are assessment of lactase activity in jejunal biopsies, a test for genetic defects [12,13], and registration of symptoms after intake of lactose [14]. Recently, the Rome Consensus Conference published a review of the methodology and indications of H2-breath testing in gastrointestinal diseases [15]. No "gold standard" is available for the diagnosis of LM and breath tests with measurement of volatile compounds and other gases (mainly CH4) were encouraged [15]. There are few comparisons between the tests and no agreement upon which one is the best.

The aims of this study were to assess the agreement between commonly used diagnostic methods for LM (breath test, s-glucose and symptoms), describe the diagnostic properties of the methods and establish the best method and cut-off levels for clinical use.

## Methods

### Subjects

Patients with intolerance to milk or dairy products and/or unexplained abdominal discomfort consistent with LM seen in one outpatient gastroenterological unit were invited to participate in the study. Exclusion criteria were age below 18 years, insufficient understanding of written instructions, intake of antibiotics the last two weeks and a previous diagnosis of LM. Age, sex, Body Mass Index (BMI), ethnicity, smoking habits, the patients' reaction to milk and dairy products, and current and past diseases were noted.

### Methods

The initial diagnostic test for LM was a combined breath- and s-glucose test after intake of 25 g lactose. The test was performed at the hospital in the morning. Prior to the test the patients were on a low fibre diet for 48 hours, and they were not allowed to eat, drink or smoke the last 12 hours before the test. Physical activity, except for the limited activity necessary to reach the hospital, was prohibited in the morning before the test. H2 and CH4 were measured in the expiratory air before, and 30, 60, 120, and 180 minutes after intake of lactose with a stationary gas chromatograph (SC MicroLyzer, QuinTron Instrument Co, Milwaukee, Wisc., USA). Alveolar air was collected using a Y-piece device (QuinTron AlveoSampler) after 10 seconds' expiration, analysed immediately after the collection and corrected for alveolar CO2. Samples for s-glucose were taken before, and 15, 30, 60, and 90 minutes after intake of lactose. An increase in H2 ≥20 ppm (parts per million) compared to the lowest measured value, an increase in CH4 ≥12 ppm compared to the value measured before the intake of lactose, or an increase in the sum of H2 and CH4 ≥15 ppm was considered as a diagnostic test for LM, as was increase in s-glucose < 1.1 mmol/L from baseline. If the s-glucose test and the breath test had concurrent outcomes (both were normal or abnormal), the patient was classified as not having LM or having LM.

Twenty-four hours after the first combined breath- and s-glucose tests the patients filled in a questionnaire which was returned to the hospital. They were asked about the presence of abdominal symptoms during and for 24 hours after the combined test. If abdominal symptoms were present, they answered three additional questions: 1) When did the symptoms start? (during the test or 0-2 h, 2-4 h, 4-8 h, > 8 h after the test) 2) What type of symptoms have you experienced? (abdominal pain/discomfort, borborygmi, bloating, diarrhoea, or constipation) and 3) How long did the symptoms last? (0-2 h, 2-4 h, 4-8 h, >8 h). In accordance with a previous study, symptoms starting within 5 h after intake of lactose and lasting for more than 2 h were classified as "Early and Long Lasting" (ELL) [2].

In cases of discrepancies between the initial breath test and the s- glucose test, the combined test was repeated without registration of symptoms, and a diagnostic gene test (Lactase C-13910T, chromosome 2q21-22) was added [12,13]. The gene tests were analyzed at the Hormone Laboratory, Aker University Hospital, Oslo, Norway. In patients with discrepancies between the breath- and the s-glucose test at the first combined test, a total of five tests were available: Two sets of breath tests, two sets of s-glucose tests and one gene test. The presumed correct diagnosis was based on an evaluation of all tests. If three or more tests were abnormal, it was concluded that the patient had LM.

Because the synthesis of CH4 consumes large amounts of H2, an increase in CH4 in the expiratory air reduces the production of H2. Therefore, the diagnostic properties of the increase in H2 + CH4x2 were studied in addition to the conventional variables (H2, CH4, H2+CH4, s-glucose and symptoms). The diagnostic properties of the gene test are not reported because of the limited number of analyses. The results of all tests (the first and the repeated ones) were used for evaluation of the diagnostic properties of the test variables.

In search of optimal cut-off levels for screening purposes, high sensitivity and therefore a lower specificity, was preferred, with further diagnostic examinations in subjects with positive tests.

### Statistical analyses

Comparisons between the groups were analysed with Mann-Whitney test and Fisher's exact test, and agreement with kappa statistics. Receiver Operating Characteristic (ROC) curves describe the diagnostic properties of the variables. SPSS version 14.0 with exact tests was used for the analyses. P-values < 0.05 were considered statistically significant, and 95% confidence intervals (CI) were calculated for the main variables.

### Ethics

The study was conducted according to the Declaration of Helsinki, and approved by the Regional Committee for Medical Research Ethics, Trondheim, Norway and Norwegian Social Science Data Services. Written informed consent was given by all participants before inclusion.

## Results

Sixty patients were included. Five patients had LM and 45 had not after the first combined breath- and s-glucose test (both tests were clearly positive or negative). In ten patients with discrepancies between the blood- and breath tests, the combined test was repeated and the gene test performed. Two of these ten patients were classified as having LM (they had three positive and two negative tests) and eight were normal (four patients had two positive and three negative tests, and four patients had one positive and four negative tests). The gene test was positive in one of the two patients with LM, and negative in eight patients without LM. In total, seven patients (12%) were classified as having LM.

Table 1 gives the characteristics of patients with and without LM. Except for a lower BMI in patients with LM, there were no significant differences between the groups. Four patients had organic diseases in clinical remission (three had ulcerative colitis and one Crohn's disease), none of them had LM. Fifty-six patients had functional bowel disorders. In patients with and without LM the increase in H2 (mean and range) were 59 ppm (0-170) and 5 ppm (0-52) respectively, increase in CH4 were 3 ppm (0-13) and 1 ppm (0-20) respectively, increase in H2 + CH4 were 62 ppm (13-170) and 6 ppm (0-52) respectively, increase in H2 + CH4x2 were 65 ppm (18-170) and 7 ppm (0-52) respectively, and increase in blood glucose were 0.7 mmol/L (0.1-1.5) and 2.0 mmol/L (0.7-4.6) respectively.

**Table 1:** Characteristics of the patients with and without lactose malabsorption (LM).

Patients' characteristics	LM	Not LM	Statistics
Number of subjects	7	53	
Male	1/7 (14%)	16/53 (30%)	ns (p = 0.66)
Age in years	46 (35-55)	36 (18-71)	ns (p = 0.15)
Scandinavian origin	7/7 (100%)	52/53 (98%)	ns (p = 1.00)
Body Mass Index (BMI)	21.1 (20.1-27.3)	24.8 (18.5-37.2)	p = 0.027
Organic bowel disease	0/7 (0%)	4/53 (8%)	ns (p = 1.00)
Abdominal discomfort after intake of milk/dairy products	4/6 (67%)	43/51 (84%)	ns (p = 0.28)
Daily smoker	1/7 (14%)	17/53 (32%)	ns (p = 0.66)

The results are given as number of patients (percentage in brackets) or median (range in brackets).

One patient did not fill in the symptom questionnaire after the lactose test, and one patient did not report exact indication of time, which made evaluation of ELL impossible. Table 2 gives the symptoms in the groups with and without LM. Presence of any ELL symptom had the best diagnostic properties, and pain/discomfort-ELL, borborygmi-ELL, and bloating-ELL were all statistically significantly related to LM.

**Table 2:** Symptoms after intake of lactose in patients with and without lactose malabsorption (LM).

Symptoms	LM (N = 7)	Not LM (N = 52)	Statistics
Pain/discomfort all	5/7 (71%)	21/52 (40%)	p = 0.223
Pain/discomfort-ELL	5/7 (71%)	8/52 (15%)	p = 0.004
Borborygmi all	5/7 (71%)	25/52 (48%)	ns (p = 0.424)
Borborygmi-ELL	5/7 (71%)	8/51 (16%)	p = 0.005
Bloating all	5/7 (71%)	24/52 (46%)	ns (p = 0.254)
Bloating-ELL	5/7 (71%)	8/51 (16%)	p = 0.005
Diarrhoea all	4/7 (57%)	16/52 (31%)	ns (p = 0.213)
Diarrhoea-ELL	3/7 (43%)	4/51 (8%)	p = 0.032
Constipation all	0/7 (0%)	4/52 (8%)	ns (p = 1.00)
Constipation-ELL	0/7 (0%)	4/52 (8%)	ns (p = 1.00)
Any symptom	6/7 (86%)	32/52 (62%)	ns (p = 0.40)
Any symptom-ELL	6/7 (86%)	11/51 (22%)	p = 0.002

ELL (= "Early and Long Lasting"): Symptoms starting within 5 h after intake of lactose and lasting for more than 2 h. Any symptom-ELL = Any "Early and Long Lasting" symptom.

The results are given as number of patients with proportions in brackets.

Table 3 gives the results of the receiver operating characteristics curves (ROC) analyses. The breath test with measurement of H2 + CH4x2 was the best one. The best cut-off levels (normal values) for the increase in H2+CH4x2, H2+CH4, H2, and s-glucose were considered to be < 18 ppm, <17 ppm, <16 ppm, and > 0.9 mmol/L respectively. Table 4 gives the agreement between the test variables with these cut-off levels. With this cut-off level, the sensitivity of H2+CH4x2 was 100%.

**Table 3:** The results of the receiver operating characteristics curve (ROC) analyses for the test variables.

Test variables	Area under ROC-curve	95% CI	Statistics
H2+CH4x2	0.976	0.945-1.007	p < 0.001
H2+ CH4	0.969	0.931-1.007	p < 0.001
s-glucose	0.924	0.836-1.013	p < 0.001
H2	0.872	0.688-1.055	p < 0.001
CH4	0.638	0.424-0.851	ns (p = 0.185)

**Table 4:** Agreement between the test variables with the best cut-off values.

Variables	H2 +CH4x2 <18 ppm	H2 + CH4 <17 ppm	H2 < 16 ppm	s-glucose > 0.9 mmol/L
H2 + CH4 < 17 ppm	0.91 (p < 0.001)	-----	-----	-----
H2 < 16 ppm	0.81 (p < 0.001)	0.90 (p < 0.001)	-----	-----
s-glucose > 0.9 mmol/L	0.44 (p = 0.001)	0.50 (p < 0.001)	0.46 (p = 0.001)	-----
Any symptom-ELL	0.38 (p = 0.002)	0.43 (p < 0.001)	0.41 (p < 0.001)	0.25 (p = 0.032)

The results are given as kappa values with statistics (p-values) in brackets.

Table 5 gives the sensitivity, specificity, positive and negative predictive values, accuracy and likelihood ratio (LR+ and LR-) for the increase in gas and s-glucose with different cut-off levels (normal values) and for the presence of any symptom-ELL.

**Table 5:** The diagnostic properties (sensitivity, specificity, positive and negative predictive values, accuracy and likelihood ratios (LR)) for the variables in the study with different cut-off levels (normal values).

Variable	Normal value	Sensitivity	Specificity	PPV	NPV	Accuracy	LR +	LR-
H2 + CH4x2	<53 ppm	44.4%	100%	100%	92.4%	92.9%	Inde-finite	0.56
H2 + CH4x2	<18 ppm	100%	90.2%	60%	100%	91.4%	10.2	0
H2 + CH4	<17 ppm	88.9%	91.8%	61.5%	98.2%	91.4%	10.8	0.12
H2	<16 ppm	77.8%	93.4%	63.6%	96.6%	91.4%	11.9	0.24
s-glucose-increase	>0.9 mmol/L	77.8%	93.4%	63.6%	96.6%	91.4%	11.9	0.24
Any symptom-ELL	Absent	85.7%	78.4%	35.3%	97.6%	79.3%	4.0	0.18

The results are based on all performed tests.

## Discussion

This study clearly demonstrates the unsatisfactory agreement between commonly used diagnostic tests for LM. The major methodological problem is the lack of a "gold standard" for the diagnosis of LM. Jejunal biopsies for assessment of lactase activity is an unreliable method due to the irregular dissemination of lactase in the intestine, the available genetic test does not detect all genetic disorders related to LM and does not diagnose secondary LM, breaths tests are highly dependent on the microflora throughout the gut, and serum glucose depends on the glucose absorption and metabolism [15]. Therefore this study assesses primarily the agreement between the test variables. But because assessment of agreement between tests requires a positive or negative result of the test, we had to diagnose LM in each patient. The final diagnosis of LM was based on an overall evaluation of all tests performed in each subject. This is the only applicable method when no formal "gold standard" is available. When the diagnosis was established, the best cut-off levels (normal values) for each of the continuous variables were chosen. A high sensitivity was preferred to avoid false negative results at the expense of a lower specificity.

Lactose breath test with measurement of H2+CH4x2 was judged as the best test. It was superior to H2+CH4 because of better sensitivity and a somewhat higher area under the ROC-curve (tables 3 and 5). The sensitivity and specificity was 100% with cut-off levels (normal values) < 18 ppm and < 53 ppm respectively. Results in the range from 18 ppm to 52 ppm render further tests necessary to obtain a conclusive diagnosis. The agreement between H2 + CH4 and H2+CH4x2 was, as expected, very good because most subjects with LM predominantly produce H2, and the variables are slight modifications of each other. Nevertheless, H2 + CH4x2 seem to be preferable in clinical use and have satisfactory diagnostic properties.

Breath tests with measurement of only H2 have been judged as reliable tests for LM [16,17]. The recently published Rome Consensus Conference report states that measurement of breath CH4 excretion is not currently recommended to improve the diagnostic accuracy of the H2 breath test due to lack of evidence, and that further studies on other gases (mainly CH4) than H2 should be encouraged [15]. In this study, the agreement between H2 and any combination of H2 and CH4 was very good, but the lower sensitivity of H2 only made it inferior to the combination of H2 + CH4x2. Since about 30% of the adult population is so-called CH4-producers and the methanogenesis consumes large quantities of H2 to produce CH4, it is reasonable to measure CH4 in addition to H2. This study showed that measurement of CH4 in addition to H2 increased the diagnostic accuracy of the breath test and that H2 + CH4x2 was the best one despite the fact that the concentration of CH4 is variable both in fasting conditions and after meals [18].

S-glucose is an alternative to breath test. The agreement with the breath test was modest and clinically unsatisfactory, but the diagnostic properties (sensitivity, specificity, PPV, NPV, and LR) were identical with that of H2 (table 5). The poor agreement and identical diagnostic properties are the result of different diagnostic classification into health and disease of the two methods. Since no gold standard is at hand it is impossible to judge between them, but because of the low specificity of s-glucose and lower area under the ROC-curve we conclude in accordance with other publications that it is inferior to breath test with measurement of H2 or H2 + CH4x2 (tables 3 and 5) [17,19].

Registration of symptoms after intake of lactose has been used as a simple test for LM [14]. Evaluation of the onset, severity and duration of symptoms for 8 hours has been recommended [15]. This study shows in accordance with previous reports, that symptoms in general are highly unreliable and unfit for clinical use [2]. Symptoms questionnaires and symptom based criteria such as "Early and Long Lasting (ELL)-symptoms" have better diagnostic properties [2,16]. In this study ELL-symptoms were superior to unspecified symptoms after intake of lactose. These findings are in agreement with the Rome Consensus Conference that symptoms should be evaluated during and for some hours after the test, and that onset and duration are of importance [15]. But even ELL-symptoms showed unacceptable diagnostic properties and poor agreement with any of the other test (table 4 and 5). This fits with the clinical observation that the prevalence of perceived lactose intolerance, which is also related to visceral hypersensitivity, is significantly higher than that of LM, and that subjects with LM can consume a variable but limited amount of lactose without developing symptoms [9,11,20].

In this study the genetic test was performed in only ten subjects with discrepancies at the first combined test and the results therefore give limited information about the usability of the test. The test is probably highly indicative of lactase non-persistence in adults [12,13]. But the fact that LM might be due to other genetic abnormalities and organic disorders in the gastrointestinal tract limits the clinical utility of the test [21,22].

The selection criteria were pragmatic and based on perceived milk intolerance or symptoms judged as possible LM by the doctor. The selection was not strictly scientific, but according to everyday practice. The prevalence of LM was rather low despite the fact that most patients had symptoms related to intake of milk or lactose and were referred with suspected LM. This is in accordance with other studies in Scandinavia showing a low prevalence of LM both in the general population and in patients with FGID [2,3]. A somewhat lower BMI was the only clinical characteristic of subjects with LM, and has also been reported in other trials [23].

Performance of the breath tests varies. In this study, the tests were performed according to recently published guidelines concerning devices for breath sampling, stationary and immediate analyses, prolonged expiration and correction for alveolar CO2, use of antibiotics, diet, cigarette smoking and physical exercise [15]. However, no mouth washing was performed, and colonic clearing was not sufficiently taken into account, but was never performed in close relation to the test. A three-sample H2 breath test is favourable compared to a two-sample [24]. The five-sample test used in this trial strengthens the results. The length of the test was three hours; 4 hours have been recommended because some subjects have a slow transit [15]. In all, it is unlikely that these minor deviations from the recently published recommendations have had any significant influence on the results. Also the dose of lactose varies. Twenty-five gram lactose (equivalent to 500 mL milk), the dose used in this trial, seems reasonable and is the recommended dose [15]. This amount gives symptoms in most subjects with LM and is within the range of normal consumption [9,11,25].

Practical and correct dietary advice to patients with FGID and food intolerance is impossible without valid and reliable tests for food intolerance. Such tests are by and large missing. Patients with food intolerance often make unnecessary changes in the diet which for some result in malnutrition [6,26]. Further improvement of the diagnostic armamentarium for food intolerance is desired to improve dietary treatment.

## Conclusion

This trial shows unsatisfactory agreement between commonly used diagnostic tests for LM. The test with the best diagnostic properties was lactose breath test with 25 g lactose and measurement of the increase in the sum of H2 and CH4x2. The area under the ROC-curve was 0.976, sensitivity was 100% with a cut-off level < 18 ppm, and specificity was 100% with a cut-off level < 53 ppm. Results in the range from 18 ppm to 52 ppm render further tests necessary to obtain a conclusive diagnosis.

## Competing interests

The authors declare that they have no competing interests.

## Authors' contributions

ØH participated in the design of the study, took part in the acquisition of data, did most of the analyses and interpretation of the data and wrote the draft manuscript.

PGF was the main contributor to the concept and design of the study, took part in the acquisition of data, was responsible for analyses and interpretation of the data, and has written the final manuscript.

Both authors approved the published version.

## Pre-publication history

The pre-publication history for this paper can be accessed here:

http://www.biomedcentral.com/1471-230X/9/82/prepub
